# By promoting cell differentiation, miR-100 sensitizes basal-like breast cancer stem cells to hormonal therapy

**DOI:** 10.18632/oncotarget.2962

**Published:** 2014-12-11

**Authors:** Annalisa Petrelli, Rosachiara Carollo, Marilisa Cargnelutti, Flora Iovino, Maurizio Callari, Daniela Cimino, Matilde Todaro, Laura Rosa Mangiapane, Alessandro Giammona, Adriana Cordova, Filippo Montemurro, Daniela Taverna, Maria Grazia Daidone, Giorgio Stassi, Silvia Giordano

**Affiliations:** ^1^ University of Torino School of Medicine, Candiolo Cancer Institute-FPO, IRCCS, Str. Provinciale, Candiolo, Torino, Italy; ^2^ Department of Surgical and Oncological Sciences, Cellular and Molecular Pathophysiology Laboratory, University of Palermo, Palermo, Italy; ^3^ Fondazione IRCCS Istituto Nazionale dei Tumori, Milan, Italy; ^4^ Molecular Biotechnology Center (MBC), Department of Oncological Sciences, Center for Molecular Systems Biology, Via Nizza, University of Torino, Torino, Italy

**Keywords:** Breast cancer, basal-like, differentiation, miR-100

## Abstract

Basal-like breast cancer is an aggressive tumor subtype with a poor response to conventional therapies. Tumor formation and relapse are sustained by a cell subset of Breast Cancer Stem Cells (BrCSCs). Here we show that miR-100 inhibits maintenance and expansion of BrCSCs in basal-like cancer through Polo-like kinase1 (Plk1) down-regulation. Moreover, miR-100 favors BrCSC differentiation, converting a basal like phenotype into luminal. It induces the expression of a functional estrogen receptor (ER) and renders basal-like BrCSCs responsive to hormonal therapy. The key role played by miR-100 in breast cancer free-survival is confirmed by the analysis of a cohort of patients’ tumors, which shows that low expression of miR-100 is a negative prognostic factor and is associated with gene signatures of high grade undifferentiated tumors. Our findings indicate a new possible therapeutic strategy, which could make aggressive breast cancers responsive to standard treatments.

## INTRODUCTION

The onset and progression of malignant tumors depend on a small pool of tumor cells with biological properties similar to those of normal adult stem cells. In accordance to this cancer stem cell hypothesis, tumors are organized in a hierarchical manner and are characterized by cells that exhibit the ability to self-renew as well as to give rise to differentiated cells. The CSCs represent the apex of this hierarchy and appear to be the phenotypic and functional equivalents of normal stem cells harboring oncogenic mutations [1]. CSCs have been isolated in most human solid tumor types, suggesting their central role in tumor development, progression and recurrence [2]. The presence of a CSC pool is associated with aggressiveness and a negative prognosis in breast cancer patients. CSCs are thought to possess intrinsic resistance to current conventional therapies as compared to the bulk tumor cell population and it has been proposed that tumor recurrence is driven by this subpopulation of CSCs [3-5].

MicroRNAs (miRNAs) are small non-coding RNAs that regulate gene expression at a post-transcriptional level, thus monitoring several biological processes. Their deregulated expression contributes to cancer development and progression and can influence both the response to therapy [6] and the development of drug resistance [7, 8]. Recently, miRNAs have also emerged as critical players in the maintenance of pluripotency, control of self-renewal and cell fate [9]. Restricted miRNA patterns are expressed only in Embryonic Stem Cells (ESCs) [10, 11] and specific miRNAs regulate and are regulated by key stem cell genes [12, 13]. The importance of the miRNA pathway in the biology of stem cells has been confirmed in Dicer-1 knock-out mice, where the loss of Dicer-1 results in the depletion of the stem cell population in embryos [14]. Moreover, Dicer-1 deficient murine ESCs fail to differentiate [15]. The majority of miRNAs that are important in ESC biology are also involved in oncogenesis. This fuels the hypothesis that miRNAs could be determinant in cell stemness both in normal and in cancer stem cells [16, 17]. In line with this hypothesis, recent data provide evidence that miRNAs might connect stemness and metastasis. Indeed, some miRNAs specifically expressed in ESCs can be inopportunely expressed in cancer cells, promoting epithelial mesenchymal transition (EMT) [18, 19] and metastasis [20].

The miR-100 family of microRNAs is composed of three members, miR-100, miR-99a and miR-99b. Comparative studies indicate that miR-100 is the oldest known animal microRNA [21] and is widely expressed in vertebrates [22]. Recent data demonstrated that miR-100 is under-expressed in human ESCs compared to differentiated cells [23] and is required for proper differentiation of mouse ESCs [24]. The role of miR-100 in cancer is quite contradictory, since it can behave either as an oncogene or as a tumor suppressor gene, depending on the tumor type [25-27].

The present work shows that miR-100 plays a pivotal role in regulating the transition between stemness and differentiation of Breast Cancer Stem Cells (BrCSCs). The ectopic expression of miR-100 in CSCs isolated from breast cancer specimens impaired their self-renewal and tumor-initiating ability. Notably, miR-100 induced luminal differentiation in basal-like BrCSCs and rendered them sensitive to endocrine therapies, such as tamoxifen and fulvestrant.

## RESULTS

### MiR-100 down-regulation induces a mammosphere-like phenotype in breast cancer cells

**Figure 1:**
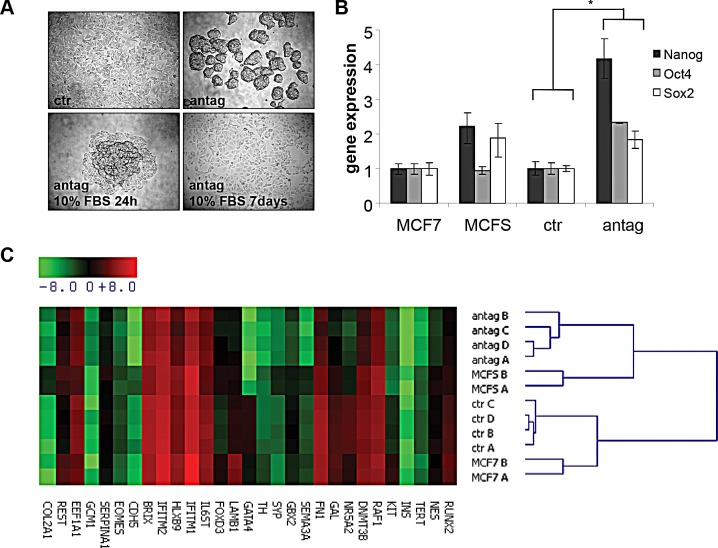
MiR-100 inhibition induces a stem-like phenotype in breast cancer cells A, phase contrast images of MCF7 cells transiently transfected with a control (ctr) or a miR-100 specific antagomir (antag). Following miR-100 antagomir transfection, obtained mammospheres retained the ability to differentiate when cultured in DMEM 10% Foetal Bovine Serum (antag 10%FBS 24h; antag 10%FBS 7 days). Magnification 4x. B, stem cell transcription factors expression in control and antagomir transfected cells, analyzed by quantitative RT-PCR. Data are average ± SD of biological replicates. MCF7 cells and mammospheres obtained from MCF7 cells upon growth in stem cell conditions (MCFS) were used as controls. * P< 0.05. C, stemness and pluripotency gene expression profiling of the cells described in (B) performed using TaqMan gene expression arrays. Gene expression is reported as −ΔCT (CT gene – CT GAPDH) median-centered. A, B, C and D indicate biological replicates.

Expression profiling studies showed that miR-100 is deregulated in various types of cancers [25-27]. Here, attention was focused on human breast cancer, where the biological role of miR-100 in tumor onset and progression remains elusive. The aim was to modulate miR-100 expression *in vitro* in breast cancer cells and study the biological consequences. The breast cancer cell line MCF7 was transiently transfected in the absence of serum, either with a miR-100 specific antagomir or a control antagomir. MiR-100 antagomir transfected cells acquired a mammosphere-like phenotype. These mammospheres retained the ability to differentiate when cultured in the presence of serum, acquiring an adherent shape (Fig. 1A). In order to ensure that antagomir-induced mammospheres showed stem cell characteristics, we analyzed the expression of the stem cell transcription factors Nanog, Oct4 and Sox2. As shown in Fig. 1B, miR-100 depleted cells expressed higher levels of the three transcription factors, compared to cells transfected with the control antagomir and to mammospheres obtained from MCF7 cells cultured in standard stem cell conditions. A wider gene expression analysis revealed that miR-100 knockdown led to a global gene reprogramming that could be responsible for the acquisition of the stem-like phenotype (Fig. 1C). Also employed was a complementary approach, evaluating miR-100 expression in mammospheres generated from breast cancer cell lines cultured in standard stem cell conditions. Consistently, the expression of the miRNA was lower in mammospheres than in the original adherent cells (Supplementary Fig. 1A,B).

### Analysis of miR-100 expression in Breast Cancer Stem Cells

**Figure 2:**
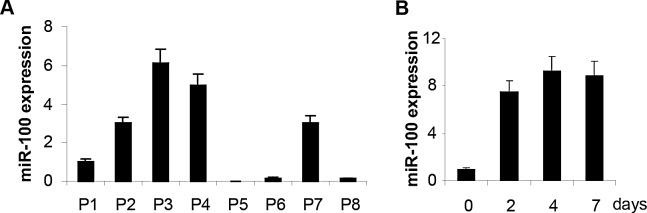
MiR-100 expression increases upon basal-like Breast Cancer Stem Cell (BrCSC) differentiation A, miR-100 expression in BrCSCs derived from human breast tumors evaluated by TaqMan RT-PCR. MiR-100 expression is reported as fold changes compared to P1. P1-P4: luminal; P5-P8: basal-like. B, miR-100 expression in basal-like BrCSCs (P5) before and after growth in differentiation condition, at the indicated times. Data are representative of two independent experiments.

The level of miR-100 expression might be critical in maintaining stemness and in determining the transition from a stem to a differentiated status in cancer cells. When miR-100 expression was analyzed in a panel of CSCs isolated from basal-like and luminal breast cancer specimens (Supplementary Table 1), lower average levels of miR-100 were found in the CSCs derived from basal-like tumors (Fig. 2A). BrCSCs derived from patient 5 (P5), classified as basal-like subtype and expressing the lowest level of miR-100, were selected for further experiments. These cells displayed low levels also of the other two members of the miR-100 family, namely miR-99a and miR-99b (Supplementary Fig. 2A). The expression of the miRNAs in P5 BrCSCs was evaluated upon growth in conditions which favored differentiation. As shown in Fig. 2B and Supplementary Fig. 2B, the level of the miRNAs promptly increased upon differentiation.

### MiR-100 impairs self-renewing and tumor-initiating ability of BrCSCs

**Figure 3:**
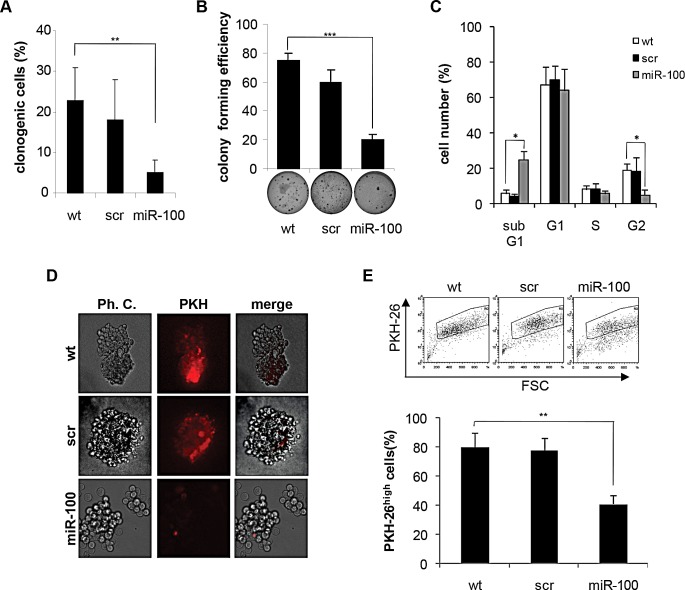
Ectopic expression of miR-100 in BrCSCs impairs self-renewal A, percentage of clonogenicity in BrCSCs (P5) wild type (wt) and stably expressing either a control scramble (scr) or miR-100. Data are average + SD of 3 independent experiments. ** P<0.01. B, colony forming efficiency of BrCSCs transduced as in (A), assessed by soft agar assay (bottom); histogram shows the quantitative analysis. Data are average + SD of 3 independent experiments. *** P<0.001. C, cell cycle analysis of wt, scramble or miR-100 stably expressing BrCSCs determined by propidium iodide staining. * P<0.05. D, representative phase contrast and fluorescence microscopy analysis of wt, scramble or miR-100 transduced BrCSCs labelled with PKH-26 and cultured in soft agar up to 40 days. E, flow cytometry analysis and quantification of PKH-26 in cells transduced as in (D), after 14 days of culture. The experiments were performed in triplicates. ** P<0.01.

In order to investigate whether miR-100 could interfere with the stem properties, an exploration of the self-renewing ability of tumor-derived P5 BrCSCs expressing stable miR-100 upon lentiviral transduction (data not shown) was undertaken. BrCSCs infected with a short hairpin scramble encoding lentivirus were used as a control. Exogenous expression of miR-100 severely impaired the clonogenic activity of BrCSCs in *in vitro* limiting dilution assay (Fig. 3A) and in the soft agar assay (Fig. 3B). Similar results were observed in the subpopulation of BrCSCs obtained by sorting the bulk population for the expression of the breast cancer stem cell markers CD49f and CD24 [28, 29] (Supplementary Fig. 3A, B). The effect of miR-100 on BrCSC proliferation was evaluated via a cell cycle analysis. These data showed a reduced G2 phase and an enlarged sub-G1 population in miRNA transduced BrCSCs as compared to corresponding controls (Fig. 3C). Consistently, an increased apoptotic rate was revealed by an enhanced caspase3/7 activity (Supplementary Fig. 3C). Labeling of BrCSCs with the lipophilic fluorescent dye PKH-26 was used to further investigate the effect of miR-100 on self-renewal. PKH-26 is retained by quiescent stem cells whereas it is gradually lost by proliferating progenitor cells [30]. MiR-100 expression reduced the percentage of PKH-26^high^ cells (Fig. 3D, E), leading to the depletion of the BrCSC proliferating pool.

**Figure 4:**
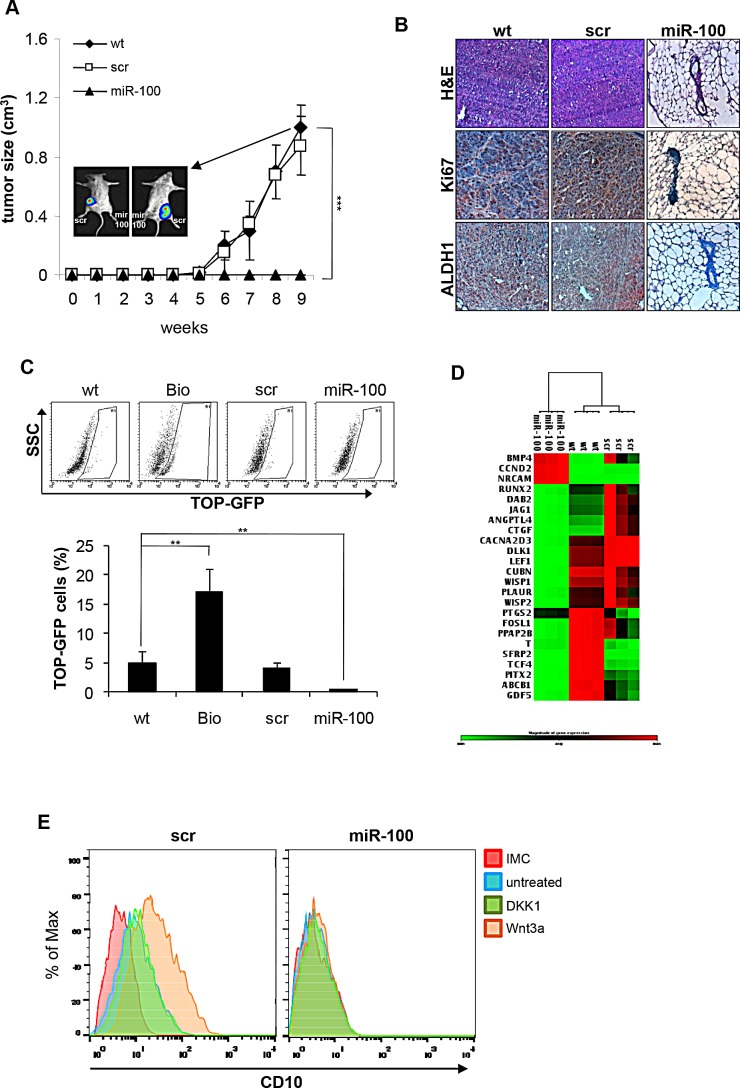
MiR-100 affects tumor-initiating ability and inhibits Wnt/β-catenin signaling pathway in BrCSCs A, *in vivo* growth of BrCSCs expressed as volume of orthotopic tumors generated by fat-pad injection of either wt, scramble or miR-100 expressing BrCSCs. Insert: dorsal and ventral whole body *in vivo* imaging analysis of orthotopic tumor growth. Data are average ± SD of experimental groups containing 6 mice. *** P<0.001. B, representative Hematoxylin/Eosin (H&E) and immunohistochemical stainings (Ki67, ALDH1) of tumors originated from wt, scramble and miR-100 BrCSCs. Magnification 20x. C, percentage of TOP-GFP positive cells in wt, scramble and miR-100 transduced BrCSCs assessed by flow cytometry. As a positive control to monitor β-catenin activity, TOP-GFP reporter lentivirus-transduced BrCSCs were treated with Bio (Bio), an inhibitor of GSK-3α/β. D, heat-map showing hierarchical clustering of genes in cells transduced as in (A). Gene expression was assessed by using a Wnt target array. E, Percentage of CD10 Max fluorescence intensity in scramble or miR-100 expressing BrCSCs, untreated or stimulated either with Wnt3a or DKK1 assessed by flow cytometry.

CSCs are defined as those cells able to originate the tumor and recapitulate the heterogeneity of the original tumor mass when implanted in immunocompromised mice. This inherent tumor-initiating capacity of CSCs is believed to be responsible for tumor relapse in patients. To address whether miR-100 could affect tumorigenic potential, miR-100 or scramble transduced BrCSCs were allowed to orthotopically grow in the mouse mammary gland of NOD/SCID mice. Interestingly, ectopic expression of miR-100 completely suppressed tumor growth (Fig. 4A). Histological examination of the fat pads showed that only a few breast cancer cells expressing the proliferation marker Ki67 and the stem cell marker Aldehyde Dehydrogenase 1 (ALDH1) were present in the residual tumor xenografts (Fig. 4B). Similar results (Supplementary Fig. 4) were obtained in an additional patient-derived basal-like BrCSC model (P8, Supplementary Table 1) displaying low levels of miRNAs of the miR-100 family (Fig 2A and Supplementary Fig. 2). These data indicate that miR-100 expression leads to the loss of CSC properties such as self-renewal and tumor-initiating ability.

### MiR-100 inhibits the Wnt signaling pathway and downregulates Polo-like kinase1 (Plk1)

The Wnt/β-catenin pathway is among the main signalling pathways involved in cancer stem cell maintenance. Tumor-initiating cells show a constitutive activation of this pathway, which can be evaluated by the LEF-1/TCF dGFP reporter [31]. In order to investigate whether the Wnt pathway contributes to miR-100 pro-differentiative program, BrCSCs wild type, scramble and miR-100 were transduced with the reporter and analyzed by flow cytometry. MiR-100 expressing BrCSCs displayed a significant reduction of β-catenin activity (Fig. 4C). Through gene array analysis, it was observed that miR-100 leads to inhibition of the Wnt signaling pathway and to downregulation of β-catenin target genes (such as WISP1/2, DLK1, TCF4 and SFRP2) that control the balance between stemness and differentiation (Fig. 4D and Supplementary Fig. 5). On the contrary, BMP4, which promotes terminal differentiation of CSCs [32], was upregulated (Fig. 4D and Supplementary Fig. 5). To evaluate if the Wnt pathway is epistatic to miR-100 in controlling breast cancer stemness, we stimulated scramble and miR-100 transduced BrCSCs and evaluated the expression of the stem cell marker CD10. As shown in Figure 4E, in control cells Wnt3a stimulation increased the expression of CD10, whereas this effect was no longer visible in cells expressing miR-100. Altogether, these data infer that miR-100 expression interferes with CSC maintenance, acting downstream to the Wnt pathway, and triggers the activation of a differentiation program.

**Figure 5:**
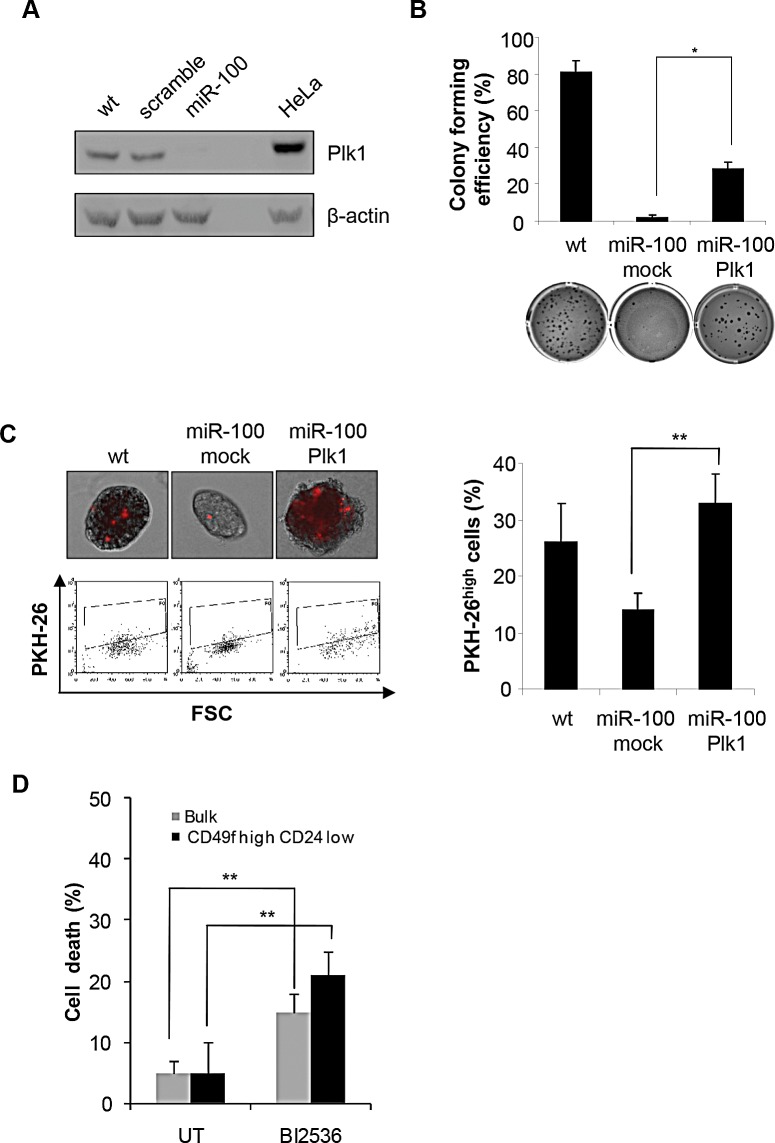
MiR-100 impairs CSC properties by down-regulating Plk1 A, Western blot analysis of Plk1 expression in wt, scramble or miR-100 transduced BrCSCs; a total protein lysate of HeLa cells was used as a positive control. B, colony forming efficiency of wt and miR-100 expressing BrCSCs transduced with either an empty vector (mock) or Plk1, assessed by soft agar assay; histogram shows the quantitative analysis. Data are average + SD of 3 independent experiments. C, representative fluorescence microscopy images of BrCSCs transduced as in (B) and labelled with PKH-26 (upper left). Flow cytometry analysis of PKH-26 in cells transduced as in (B) after 14 days of culture (bottom left) and the corresponding quantification (right). D, Analysis of BrCSC mortality in bulk and CD49f^high^/CD24^low^ sorted BrCSCs upon treatment with the Plk1 inhibitor BI2536 (10nM) for 72 hours. The experiments were performed in triplicates. UT: untreated.

In an attempt to untangle the molecular mechanisms underlying miR-100 induced phenotype, the expression of Plk1, a known miR-100 target gene recently shown to be involved in the regulation of stem cell proliferation and differentiation [33, 34], was analyzed. MiR-100 transduced BrCSCs displayed a significant reduction of Plk1 protein (Fig. 5A). Rescue experiments were performed by re-introducing Plk1 in miR-100 expressing BrCSCs and evaluating their self-renewing ability. Upon Plk1 expression, colony forming efficiency was partially recovered (Fig. 5B), while self-renewal was restored at a level comparable to wild type BrCSCs (Fig. 5C). Consistently, the Plk1 inhibitor BI2536 impaired viability both in the bulk population of BrCSCs and in the CD49f^high^/CD24^low^ sorted cells (Fig. 5D). These results indicate that Plk1 plays a key role in mediating miR-100 induced phenotype.

SMARCA5 and SMARCD1, two miR-100 targets belonging to the SWI/SNF protein family, have recently been shown to participate in differentiation of embryonal [24, 35] and cancer stem cells [36]. When we analyzed their expression, we found that these two proteins were significantly down-regulated in miR-100 transduced BrCSCs compared to controls (Supplementary Fig. 6A, B), suggesting that SMARC, together with Plk1, reduction could contribute to miR-100 dependent differentiation.

### MiR-100 promotes luminal differentiation and renders basal-like BrCSCs responsive to hormonal therapy

**Figure 6:**
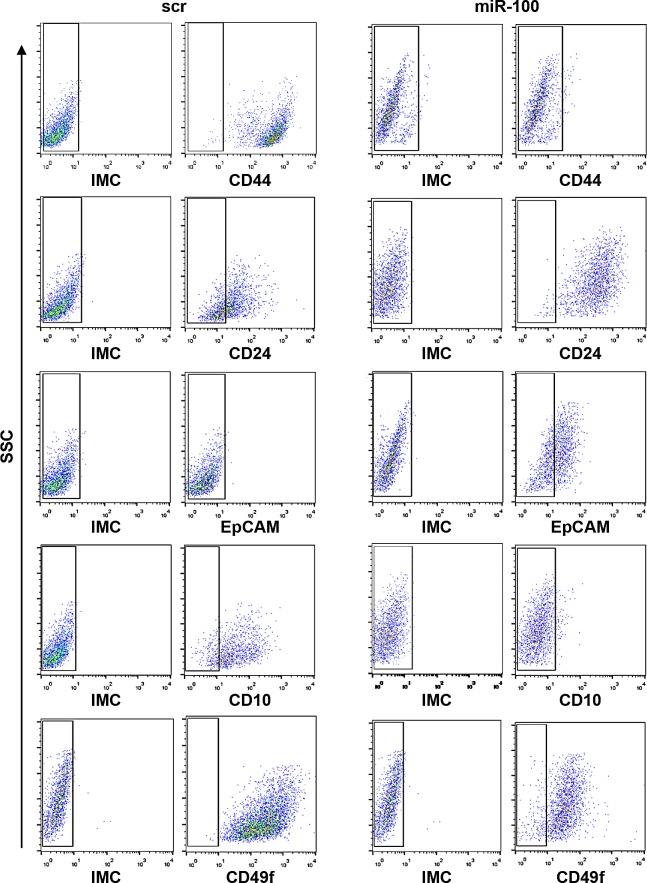
Ectopic expression of miR-100 reduces stem cell markers and induces markers of differentiation Flow cytometry analysis of CD44, CD24, CD10, CD49f and EpCAM expression in BrCSCs (P5) scramble and stably expressing miR-100. IMC: Isotype Matched Control.

**Figure 7:**
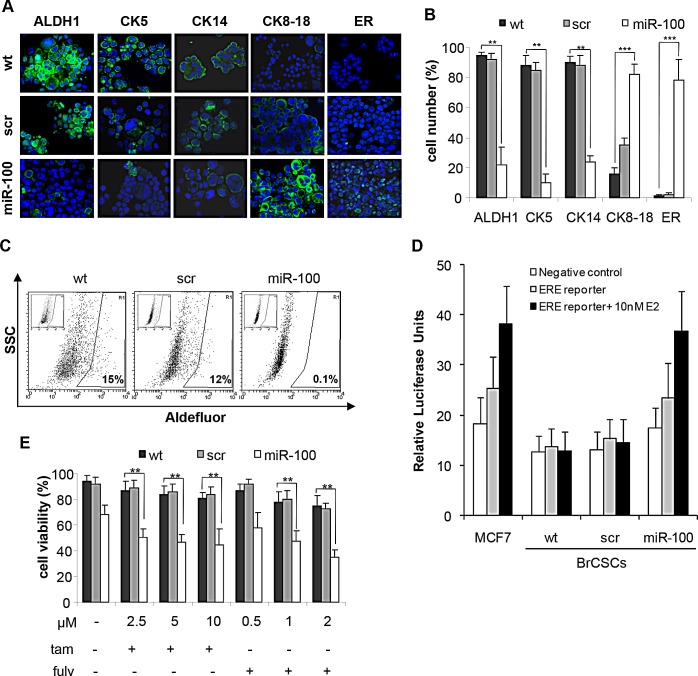
Ectopic expression of miR-100 reduces stem cell markers, promotes luminal differentiation and renders basal-like BrCSCs responsive to endocrine therapy A, representative confocal microscopy images of immunofluorescence (IF) analysis of ALDH1, Cytokeratins (CK5, CK14, CK8-18) and estrogen receptor (ER) performed in BrCSCs (P5) wt and stably expressing either a control scramble or miR-100. Nuclei were counterstained by Toto-3 (blue). Magnification 40x. B, quantification of the IF staining shown in (A), performed in three independent replicates. ** P<0.01 *** P<0.001. C, representative FACS analysis of Aldefluor assay performed in wt, scramble and miR-100 BrCSCs. Cells were exposed to Aldefluor substrate (BAAA); cells treated with the specific inhibitor of ALDH1 (DEAB) are shown in the insert panels and were used to define the population with low and high (gated region) ALDH1 activity. D, representative analysis of ER-dependent transcriptional activity. The assay was performed in wt, scramble and miR-100 BrCSCs, non transfected (negative control) or transfected (ERE-reporter) with a construct where an Estrogen Responsive Element (ERE) containing promoter drives luciferase expression. Luciferase activity was evaluated in the absence or in the presence of 10nM 17-β-estradiol (E2). MCF7 cells were used as positive control of response to estradiol. E, Analysis of BrCSC viability upon treatment with tamoxifen (tam) and fulvestrant (fulv) at the indicated doses. The experiments were performed in triplicates. ** P<0.01.

To further validate the role of miR-100 in controlling stemness and differentiation of breast cancer cells, we evaluated either by flow cytometry or immunofluorescence (IF) the expression of putative stem/progenitor and differentiation markers upon ectopic expression of the miRNA. FACS analysis showed that stem cell markers, such as CD44, CD10 and CD49f, were drastically reduced, while the differentiation markers CD24 and EpCAM increased (Fig. 6). We also assessed the expression of additional mammary stem/progenitor markers by immunofluorescence analysis. Early progenitor/stemness markers such as ALDH1, Cytokeratin 5 and myoepithelial Cytokeratin 14 were reduced in miR-100 transduced BrCSCs; conversely, the luminal epithelial markers Cytokeratin 8-18 and ER were *de novo* expressed (Fig. 7A, B and Supplementary Fig. 7A, B). Expression of ER upon miR-100 transduction was confirmed by FACS analysis as well (Supplementary Fig. 8A). Using the Aldefluor assay, we found that ALDH1 activity was also greatly reduced (Fig. 7C and Supplementary Fig. 7C). Furthermore, the luminal differentiation promoted by miR-100 was observed in CD49f^high^/CD24^low^ sorted BrCSCs as well (Supplementary Fig. 8B), confirming that miR-100 not only interferes with stemness maintenance, but also converts the breast cancer phenotype from basal to luminal-like.

Then, we wondered whether the ER pathway was functional in miR-100 expressing BrCSCs. Indeed, cells transfected with an ERE luciferase reporter displayed an increased luciferase activity in the presence of miR-100 compared to control cells (Figure 7D). Finally, we investigated whether miR-100 expression could sensitize basal-like unresponsive BrCSCs to ER inhibitors. Viability of miR-100 transduced P5 and P8 BrCSCs was significantly affected by tamoxifen and fulvestrant, at concentrations comparable to those used as optimal dose regimen for the treatment of hormone receptor positive breast cancers (Fig. 7E and Supplementary Fig. 7D). These findings indicate that the differentiation program activated by miR-100 is able to induce ER expression and to sensitize basal-like BrCSCs to endocrine therapies.

### Low miR-100 expression predicts poor prognosis in breast cancer patients

**Figure 8:**
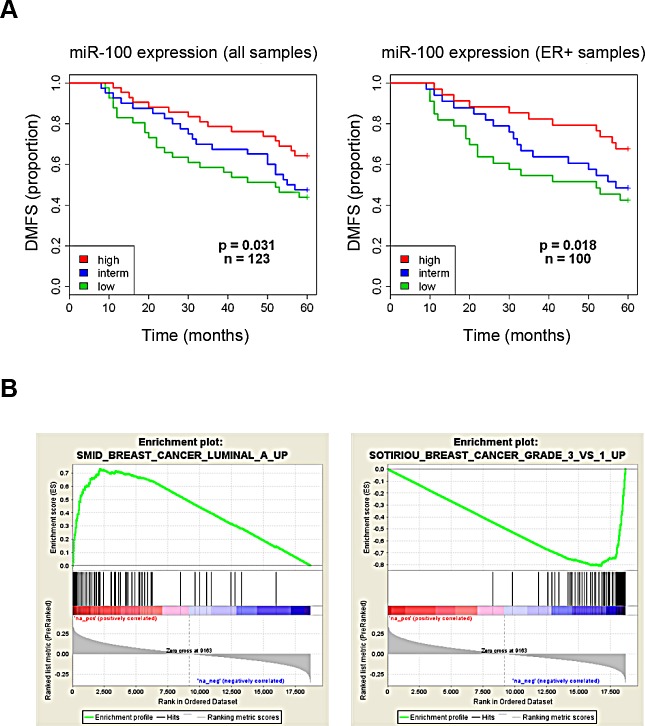
Low expression of miR-100 correlates with poor prognosis and high grade tumor signatures in breast cancer patients A, Kaplan-Meier curves associated to miR-100 expression in a cohort of 123 breast cancer patients (for characteristics of patients see Supplementary Table 2). Left panel: all patients; right panel: estrogen receptor-positive patients. B, miR-100 expression was correlated with gene expression data and gene set enrichment analysis was performed on ranked genes. Two of the top positively (left) or negatively (right) correlated gene sets are reported.

To understand the clinical relevance of miR-100 as a possible prognostic factor, we examined its expression in 123 breast tumor specimens. Patients underwent radical local-regional therapy for resectable node-negative breast cancer and received no further adjuvant treatments until relapse (Supplementary Table 2). Patients were categorized according to tertiles of miR-100 expression. At a median follow-up of 60 months, low miR-100 expression was associated with worse distant metastasis-free survival in the whole population and in the subgroup with ER-positive tumors (Fig. 8A). To confirm the prognostic value of miR-100, a univariate Kaplan-Meier analysis was conducted on two validation sets of breast cancer patients who underwent surgery and received adjuvant treatments (GEO dataset superSeries GSE22220 and Supplementary Table 3). MiR-100 expression could stratify patients according to different prognosis (Supplementary Fig. 9) in these case series, as well. Moreover, as highlighted by Gene Set Enrichment Analysis (GSEA), miR-100 positively correlated with genes expressed in luminal tumors. On the other hand, low levels of the miRNA were associated with gene signatures of high-grade, poorly differentiated cancers (Fig. 8B). This confirms that low miR-100 expression is related to a more undifferentiated phenotype.

## DISCUSSION

Studies performed over the past years have strengthened the hypothesis that breast tumors originate from mammary stem cells, as a consequence of dysregulation in the usually tightly controlled process of self-renewal. Despite the fact that these CSCs represent a small percentage of the tumor mass, growing evidence points to them as being the cells responsible for the life-threatening terminal evolution of the disease. They are resistant to conventional therapies and can escape anti-cancer treatments, giving rise to relapse in patients [3, 4, 37]. Therefore, untangling the molecular mechanisms underlying CSC maintenance becomes a priority for the development of new cancer therapies able to eradicate the disease.

It is known that miRNAs contribute in sustaining stemness of embryonic stem cells, since ESC maintenance is hampered by deficiency in the miRNA processing [14]. At the same time, miRNAs are essential regulators of ESC differentiation, which is associated with changes in the expression of specific miRNA patterns [38]. An important role of miRNAs in controlling self-renewal and differentiation of cancer stem cells has also recently been described [15, 39, 40].

Breast cancer is a heterogeneous disease which includes distinct types of tumors characterized by different histological origins, molecular features and prognosis [41]. Luminal tumors are characterized at the molecular level by the expression of ER and PR receptors. ER and PR signaling sustains tumor growth and thus, therapies interfering with ER activation are currently the gold standard for the treatment of this type of cancer. Basal-like tumors are defined by their lack of ER, PR and HER2 expression in about 75% of cases. These cancers are poorly differentiated and are loaded with CSCs, a feature that is associated with a poor clinical outcome [28, 42]. The absence of the specific molecular targets in basal-like breast cancers renders ineffective the targeted therapies that significantly improve prognosis for hormone receptor-positive and HER2-overexpressing breast cancers. Therefore, a possible therapeutic strategy to treat basal-like tumors is to induce CSC differentiation and to allow the expression of genes, such as ER, to be used as targets. The strategy of using drugs that force malignant cells to terminally differentiate has been known for the past thirty years as “differentiation therapy” and is strictly connected to the concept of tissue-selective therapy [43]. Such an approach drastically reduces side effects in patients since it avoids indiscriminately killing proliferating cells and instead concentrates its efficacy on cancer cells in a tissue specific manner, taking advantage of differentiation molecules that are specifically expressed in the selected tissue. This therapy has its successful paradigm in the treatment of acute promyelocytic leukemia (APL) with all-trans retinoic acid [44]. However, despite the promising results obtained in hematological malignancies, the application of differentiation therapy in solid tumors has been hampered due to inadequate knowledge of the mechanisms governing cell differentiation. The current findings uncover miR-100 as a key player in the complex scenario of the differentiation process. They show that miR-100 is critical in controlling stemness and differentiation of patient-derived basal-like breast CSCs. MiR-100 interferes with the CSC properties, impairing self-renewal and blocking tumor-initiating ability.

Our results also suggest that miR-100 induces a pro-differentiative program which involves the Wnt/β-catenin pathway. Activation of β-catenin and expression of Wnt target genes are involved in the balance between stemness and differentiation. Indeed, it was found that Wnt pathway activation was reduced in cells expressing miR-100 and Wnt3a treatment was unable to promote the expression of stem cell markers. Moreover, the down-regulation of Plk1, a serine/threonine kinase target of miR-100 that controls cell cycle progression, was observed. Recently, Plk1 has been implicated in the regulation of stem cell maintenance and proliferation, as inhibiting Plk1 activity impaired growth and induced apoptosis of neurospheres [34] and of colon cancer initiating cells [33]. Here it was shown that Plk1 is required to sustain expansion of BrCSCs, as miR-100 mediated down-regulation of Plk1impairs CSC properties and depletes the CSC pool.

Furthermore data from this research provide evidence that the exogenous expression of this miRNA not only hampers the replication potential of basal-like BrCSCs, but also induces their differentiation toward a luminal phenotype. In this way, miR-100 allows the conversion of an aggressive molecular subtype of cancer into a subtype with a better prognosis, for which effective treatments are available. Indeed, miR-100 promoted the expression of the estrogen receptor and rendered basal-like cells responsive to 17-β-estradiol and to the anti-proliferative activity of tamoxifen and fulvestrant. Several miRNAs (such as miR-206, miR-221/222, miR-22, miR-17/92 and miR-145) inhibit ER expression and sustain resistance to hormonal therapies [45-47]. However, miRNAs that are able to induce *de novo* expression of this target molecule and responsiveness to endocrine treatment in triple negative breast cancer cells have never been previously described.

The results obtained in this pre-clinical system are consistent with the analysis performed on breast tumor specimens. High levels of miR-100 are associated with luminal gene signatures, while undifferentiated high grade tumors correlate with lower miRNA expression. This is also in line with recent findings that demonstrate the presence of a higher CSC content in G3 poorly differentiated tumors [30]. This could explain the poor outcome and the tendency to relapse [28]. Moreover, the analysis of breast tumor specimens from two validation data sets, which include patients who received adjuvant therapy, revealed that low expression of miR-100 correlates with a negative prognosis in ER-positive patients. This raises the question as to whether this miRNA might be a predictor of hormonal therapy response. Further studies on appropriate cohorts of patients are warranted to address this hypothesis.

Overall, these findings have relevant clinical implications, as they suggest the possibility of targeting basal-like CSCs using a pro-differentiative therapy approach, by promoting miR-100 expression. Moreover, they propose miR-100 as a response predictor to endocrine therapy and offer a therapeutic perspective in treating an aggressive tumor type, for which there are no effective therapeutic options available at this time.

## METHODS

### Tissue collection and cell culture

Fresh tissue acquired from mastectomies of 8 patients (age 40–89) were collected at the University of Palermo and Fondazione IRCCS INT of Milan, in accordance with ethical standards. Breast tumor cells were purified from fresh tissue via enzymatic digestion as previously described [48]. Thereafter, single cell suspensions were plated in ultra-low attachment flasks (Corning) at a density of 1×10^5^/ml and grown in a medium supplemented with bFGF (10 ng/ml, Sigma) and EGF (20 ng/ml, Sigma). To induce differentiation, cells were cultured in adherent conditions in Ham's/F-12 medium (Euroclone), supplemented with 5% fetal bovine serum (FBS), insulin (25μg/ml, Sigma) and hydrocortisone (1mg/ml, Sigma). MCF7 cells were purchased from ATCC and cultured in DMEM supplemented with 10% fetal bovine serum (Sigma).

### Cell transfection, lentiviral constructs and cell transduction

MCF7 cells were transiently transfected with a miR-100 specific antagomir (AM10188, Ambion) in serum free medium using Lipofectamine 2000 (Invitrogen). MiR-100 stable expression was obtained by transducing cells with a lentivirus expressing miR-100 as previously described [26]. The lentiviral short hairpin scramble (pLKO.1) used as a control was purchased from Sigma. For the *in vivo* imaging, gene transfer was performed using a TWEEN lentiviral vector containing luciferase (LUC) and green fluorescent protein (GFP) as reporter genes. For β-catenin activity assay, BRCSCs were transduced with lentiviral-TOP-dGFP-reporter (Addgene) that consists of a LEF-1/TCF-responsive promoter upstream d2-eGFP. Transfection of packaging cell line HEK-293T was performed using FuGENE Reagent (Roche) and following the manufacturer's instructions. BRCSCs were dissociated into single cells, then infected with 100 ng virus/10^5^ cells.

### Biological assays

For the clonogenic assay, cells were plated on a ultra-low adhesion 96-well plate at a concentration of a single cell per well and observed for 21 days. Wells containing either none or more than one cell were excluded from the analysis. For soft agar assay, 0.4% Seaplaque soft agar (Lonza) was diluted with stem cell medium and was covered by a second 0.3% soft agar layer in which BrCSCs were embedded. After 21 days, colonies were stained with 0.005% crystal violet (Sigma) for 1 hour at 37°C. For anti-estrogen treatment, cells were treated with 2.5-10 μM tamoxifen (Selleckchem) and 0.5-2 μM fulvestrant (Selleckchem) for 24-48 hours. Cell viability was assessed by means of a cell Titer Aqueous Assay Kit (Promega) following the manufacturer's instructions. Alternatively, cell death was evaluated by orange acridine/ethidium bromide staining as previously described [48] and using Cell Titer-Glo luminescent cell viability assay kit (Promega). For the assessment of apoptosis, 3×10^3^ cells/well were seeded in ultra-low adhesion 96-well plate in stem cell medium and activation of caspases 3 and 7 was evaluated after 72hours using the Caspase-Glo 3/7 Assay (Promega). The proliferative rate of stem cells was analyzed using the PKH-26 assay (2×10^−6^ M, Sigma) according to manufacturer's instructions. Samples were analyzed by FACSCalibur (BD Biosciences). For β-catenin activity experiments, cells transduced with the indicated lentiviral constructs were analyzed by FACS. As a positive control, cells were treated with BIO (1uM, Calbiochem) for 24 hours in order to inhibit GSK-3α/β. For viability assay, single cells suspensions were treated with the Plk1 inhibitor BI2536 (10nM, Selleckchem) for 72 hours.

In order to evaluate changes of CD10 expression upon Wnt pathway modulation, scramble or miR-100 BrCSCs were treated either with Wnt3a (300ng /ml) every 6 hours for 24 hours or DKK1 (200ng/ml) for 24 hours and then analyzed by flow cytometry.

### FACS analysis and Cell sorting

BrCSCs were exposed to primary antibodies CD44 (BU75, Ancell), CD24 (ML5, R&D System), CD10 (FR4D11, Santa Cruz Biotechnology), CD49f (GoH3 Miltenyi Biotec), EpCAM (AF960, R&D System) or corresponding isotype controls, rinsed and labeled with secondary antibodies. BrCSCs were stained with CD49f and CD24 and successively sorted via flow cytometry using an FACSAria cell sorter (BD Biosciences). The analysis of ALDH1 activity was performed using the ALDEFLUOR kit (StemCell Technologies). For cell cycle analysis, BrCSCs were fixed in 70% ethanol and incubated with 50 μg/mL propidium iodide (Sigma-Aldrich), 3.8 mmol/L sodium citrate (Sigma) and 10 μg/mL RNase (Sigma). Samples were analyzed by FACSCalibur and CellQuest Software (BD Biosciences).

### Quantitative analysis of microRNA and gene expression

Total RNA was extracted using TRI Reagent solution (Ambion) following manufacturer's instructions. Analysis of miR-100, miR-99a and miR-99b was performed starting from equal amounts of total RNA/sample (10ng) using the specific Taqman microRNA assay kits (Applied Biosystems). MiRNA expression was calculated as fold change using the delta-delta CT method and RNU48 as endogenous control.

Gene expression was evaluated by quantitative real-time PCR using the EXPRESS SYBR green (Invitrogen). Retrotranscription was performed using the High Capacity Retrotranscription Kit (Applied Biosystems) starting from 500ng of total RNA.

Expression profiling of pluripotency genes was performed starting from 1.5ug of total RNA using TaqMan Human Stem Cell Pluripotency Arrays (Applied Biosystems), according to manufacturer's instructions. Gene expression was calculated as ΔCT (CT gene - CT GAPDH). The differentially expressed genes were statistically analyzed using t-test and genes with a P<0.05 were included in the heat-map. Expression was reported as −ΔCT normalized to the median. The heat-map was obtained by Gedas software [49].

Expression of Wnt target genes was performed through RT^2^ profiler PCR array (PAHS-243, Qiagen), according to manufacturers’ instructions. Arrays were performed independently for BrCSCs wt, scamble and miR-100 and at least 3 technical replicates were run for each sample. Cycle threshold values were normalized using the average of 5 housekeeping genes on the same array (B2M, HPRT1, RPL13A, GAPDH and actin B). The comparative cycle threshold method was used to calculate the relative quantification of gene expression.

### Animal models

Orthotopic xenografts were obtained by injecting 10^5^ BrCSCs P5 or P8 in the murine mammary gland of three week-old female NOD/SCID mice. *In vivo* imaging was performed by a Biospace instrument upon i.p. injection of Luciferin (150 mg/kg, Promega).

### Immunohistochemistry

Immunohistochemistry was performed on paraffin-embedded sections and labelled with Ki67 (MIB-1, Dako) and ALDH1 (44, BD Biosciences). Immunocomplexes were revealed by peroxidase labeled streptavidin following manufacturer's instructions (LSAB2, Dako). Stainings were revealed using AEC (Dako) and counterstained with aqueous hematoxylin.

### Immunofluorescence

BrCSCs were fixed with 2% paraformaldehyde for 30 minutes at 37°C and incubated O.N. at 4°C with the following primary antibodies: ALDH1 (44, BD), CK5 (XM26, Novocastra), CK14 (LL002, Novocastra), CK8-18 (CD10, Novocastra), MUC1 (BD Pharmigen), VIMENTIN (R28, Cell Signaling), ER (6F11, Novocastra). Thereafter, cells were labeled with secondary antibodies (Invitrogen). Nuclei were counterstained with Toto-3 (Invitrogen).

### Western blot

Cell pellets were lysed in buffer (TPER, Pierce; 300 mM NaCl; 1 mM orthovanadate; 200 mM PEFABLOC, Roche; 5 μg/ml Aprotinin, 5 μg/ml Pepstatin A, 5 μg/ml Leupeptin, Sigma). Lysates (30 μg/lane) were fractioned with SDS-PAGE and blotted to PVDF. Membranes were blocked with no-fat dry milk in TBS 0.05% Tween20 and incubated overnight with a specific antibody for Plk1 (208G4, Cell Signaling), SMARCD1 (AB81621, Abcam), SMARCA5 (MAB120, Millipore) and β-actin (JLA20, Calbiochem). Densitometric analysis was performed by UVP.

### ERE-reporter assay

Cells were transfected with 1μg mixture of an inducible ERE-responsive firefly luciferase construct (kindly provided by Dr. De Bortoli, University of Torino) and a constitutively expressing Renilla luciferase construct (40:1) (Cignal Reporter Assay, Qiagen) using Lipofectamine 3000 reagent (Life Technologies) according to the manufacturer's instructions. Cells were seeded into ultra-low attachment 24 wells plates (Corning) at a density of 100,000 cells per well. After 48 hours from transfection, cells were treated for 6 hours with either vehicle or 10 nM 17-β-Estradiol (E2758, Sigma) and the reporter activity was measured by luminescence. Values were normalized to Renilla luciferase activity; data are presented as relative luciferase values.

### Case series at INT

The case series collected at INT in Milan consisted of 123 patients with primary invasive breast cancer and negative lymph nodes. They were subjected to radical and/or conservative surgery, plus radiotherapy. These patients, recruited from 1990 to 1998, were identified among those who developed distant metastasis within 5 years of treatment (59 patients, disease free survival range: 8-58 months) and those who were disease-free for more than 60 months (64 patients, disease free survival range: 60-185 months). The two subsets of patients were comparable in age, tumor size, histotype, ER and HER2 status. Each patient wrote an informed consent, which authorized the use of material for research purposes. The study was approved by the Independent Ethics Committee and the Institutional Review Board.

### miRNA and gene expression analysis in breast cancer patients

Global gene and miRNA expression data were obtained using the HumanRef-6_v3 and Human miRNA_V2 Illumina BeadChips, respectively. Raw data were generated using the Illumina BeadStudio 3.8 software and processed using the Bioconductor *lumi* package. After quality control, the Robust Spline Normalization was applied. For gene signature expression analysis, all genes were correlated with miR-100 expression and gene sets from the C2 collection of the MSigDB were tested for their enrichment among positively or negatively correlated genes using GSEA (http://www.broadinstitute.org/gsea).

### Statistical analysis

Data were expressed as average ± standard deviation. Statistical significance was determined by the t-test or by Analysis of Variance (one-way or two-way) with Bonferroni post-test. P values <0.05 were considered significant (* P<0.05; ** P<0.01; *** P<0.001).

## SUPPLEMENTARY MATERIAL FIGURES AND TABLES


