# Correction: By promoting cell differentiation, miR-100 sensitizes basal-like breast cancer stem cells to hormonal therapy

**DOI:** 10.18632/oncotarget.27151

**Published:** 2019-08-13

**Authors:** Annalisa Petrelli, Rosachiara Carollo, Marilisa Cargnelutti, Flora Iovino, Maurizio Callari, Daniela Cimino, Matilde Todaro, Laura Rosa Mangiapane, Alessandro Giammona, Adriana Cordova, Filippo Montemurro, Daniela Taverna, Maria Grazia Daidone, Giorgio Stassi, Silvia Giordano

**Affiliations:** ^1^ University of Torino School of Medicine, Candiolo Cancer Institute-FPO, IRCCS, Str. Provinciale, Candiolo, Torino, Italy; ^2^ Department of Surgical and Oncological Sciences, Cellular and Molecular Pathophysiology Laboratory, University of Palermo, Palermo, Italy; ^3^ Fondazione IRCCS Istituto Nazionale dei Tumori, Milan, Italy; ^4^ Molecular Biotechnology Center (MBC), Department of Oncological Sciences, Center for Molecular Systems Biology, Via Nizza, University of Torino, Torino, Italy

**This article has been corrected:** Due to errors in image assembly, the flow cytometry profiles depicting the isotype matched control (IMC) for CD49f on both scramble (scr) and miR-100 reported in Figure 6 are incorrect. Additionally, we noticed that the IMC reported for CD24 and CD10 in miR-100 are the same. Being both CD24 allophycocianin (APC) and CD10 APC IgG1, they should have the same IMC, as already reported. The corrected Figure 6 is shown below. The authors declare that these corrections do not change the results or conclusions of this paper.

Original article: Oncotarget. 2015; 6:2315–2330. 2315-2330 . https://doi.org/10.18632/oncotarget.2962

**Figure 6:**
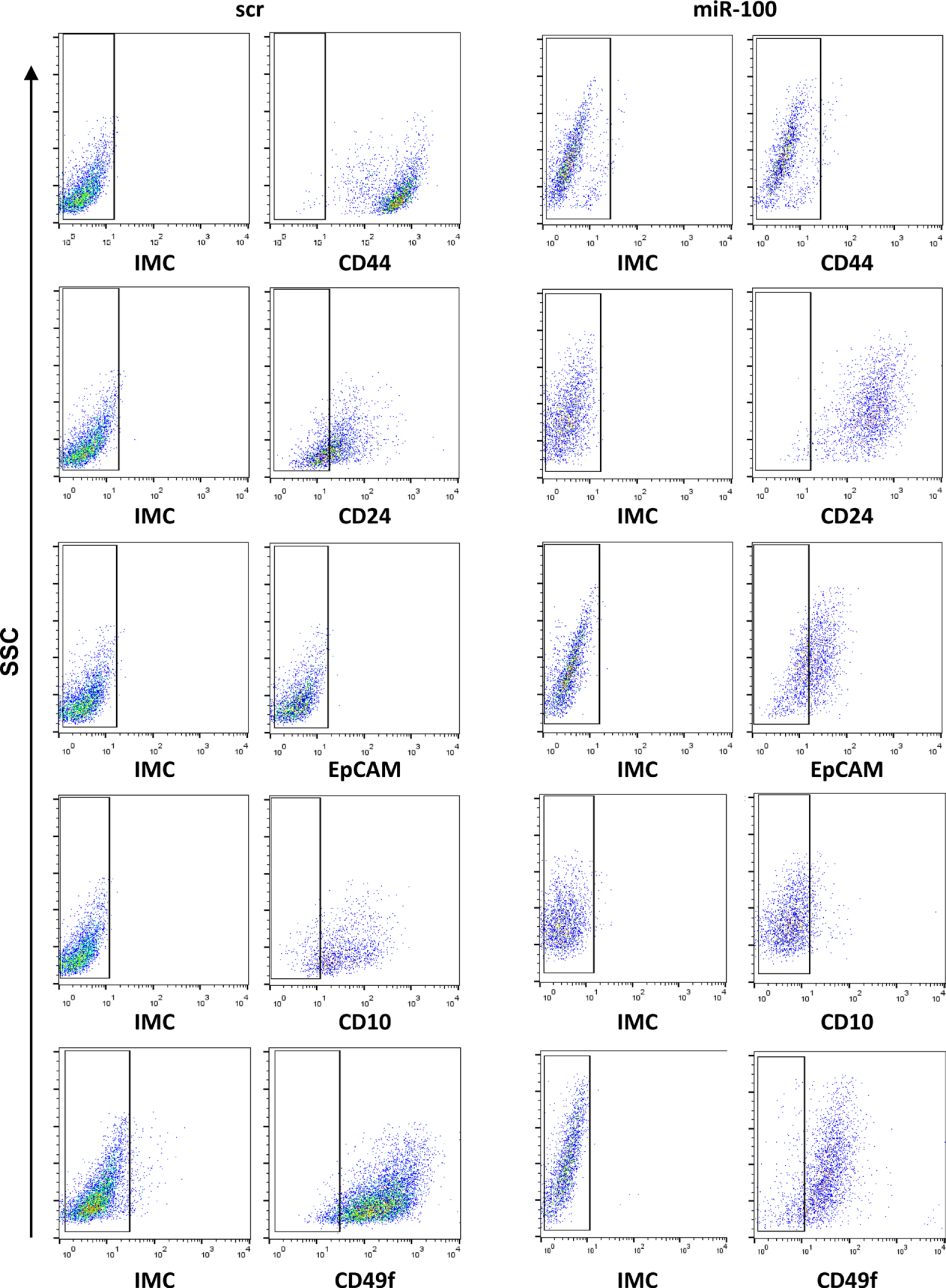
Ectopic expression of miR-100 reduces stem cell markers and induces markers of differentiation. Flow cytometry analysis of CD44, CD24, CD10, CD49f and EpCAM expression in BrCSCs (P5) scramble and stably expressing miR-100. IMC: Isotype Matched Control.

