# Supplementary material for: By promoting cell differentiation, miR-100 sensitizes basal-like breast cancer stem cells to hormonal therapy
Source: Oncotarget. 2014 Dec 11;6(4):2315–30. doi: 10.18632/oncotarget.2962 (PMC4385854; doi:10.18632/oncotarget.2962)
Supplement: Supplementary file 1 [file oncotarget-06-2315-s001.pdf]

By promoting cell differentiation, miR-100 sensitizes basal-like breast cancer stem cells to hormonal therapy

Supplementary Material

MATERIALS AND METHODS

Validation case series (cohort II)

58 frozen primary invasive ductal breast cancer specimens, obtained from patients who underwent primary surgical treatment between 1988 and 2001 at a median age of 54 years (25-82), were selected from the Tumor Bank of the Department of Obstetrics and Gynecology, University of Turin. Eligibility criteria were the following: diagnosis of invasive breast cancer, all T and N stage, no distant metastasis at diagnosis, complete clinical-pathological data and updated follow up for at least 72 months and up to 100 months. All patients were treated with radical modified mastectomy or quadrantectomy and axillary dissection plus breast irradiation. High-risk node- negative and node-positive patients received adjuvant treatments (generally 6 cycles of CMF, 600 mg/m2 cyclophosphamide, 40 mg/m2 Metotrexate, 600 mg/m2 5- Fluorouracil) and/or 20 mg tamoxifen daily for 5 years in ER+ cases. ER and PR status was determined by immunohistochemical stainings, patient stage distribution was assessed as prescribed by the UICC clinical staging guidelines and tumor grading was performed according to Elston and Ellis.

Reference List

1. Buffa FM, Camps C, Winchester L, Snell CE, Gee HE, Sheldon H et al. microRNA-associated progression pathways and potential therapeutic targets identified by integrated mRNA and microRNA expression profiling in breast cancer. Cancer Res 2011;71:5635-45.

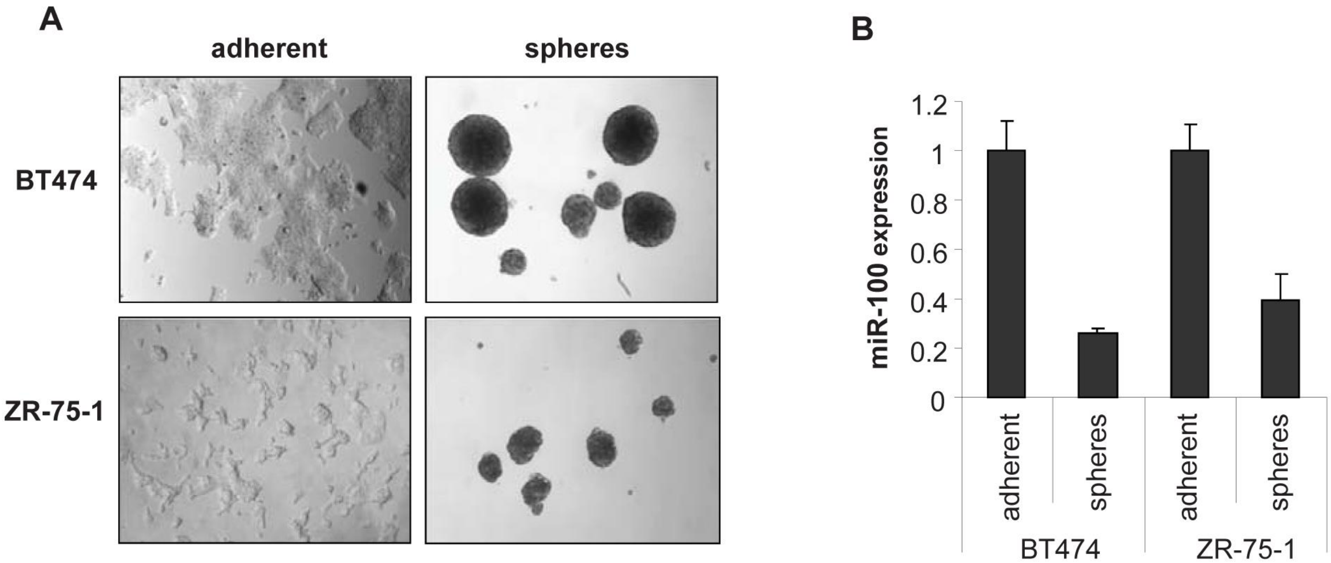

Supplementary Figure 1: MiR-100 expression is lower in mammospheres than in differentiated cells. **A,** phase contrast images of the breast cancer cell lines BT474 and ZR-75-1 grown in adherent conditions (adherent) or cultured as spheres in stem cell conditions (spheres). Magnification 4x. **B,** evaluation of miR-100 expression by TaqMan RT-PCR in the cells shown in (A), reported as fold changes compared to adherent cells. Results are representative of 2 independent experiments.

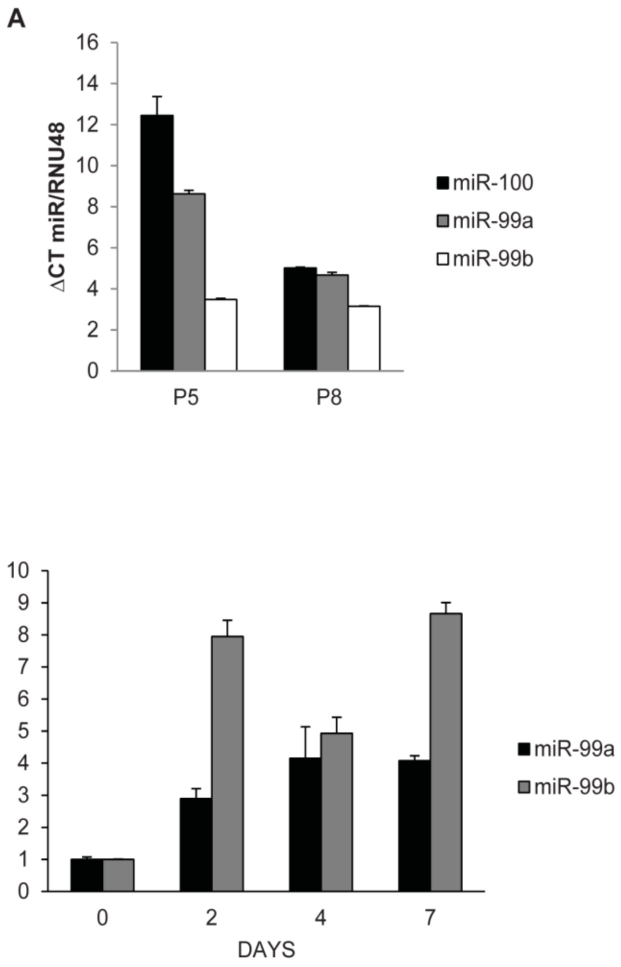

Supplementary Figure 2: MiR-100 family member expression in BrCSCs. A, miR-100, miR-99a and miR-99b expression in P5 and P8 BrCSCs evaluated by TaqMan RT-PCR and reported as ΔCT versus RNU48 endogenous control. **B,** miR-99a and miR-99b expression in P5 BrCSCs before and after growth in differentiation condition at the indicated times. Data are reported as fold change compared to undifferentiated cells (0 days).

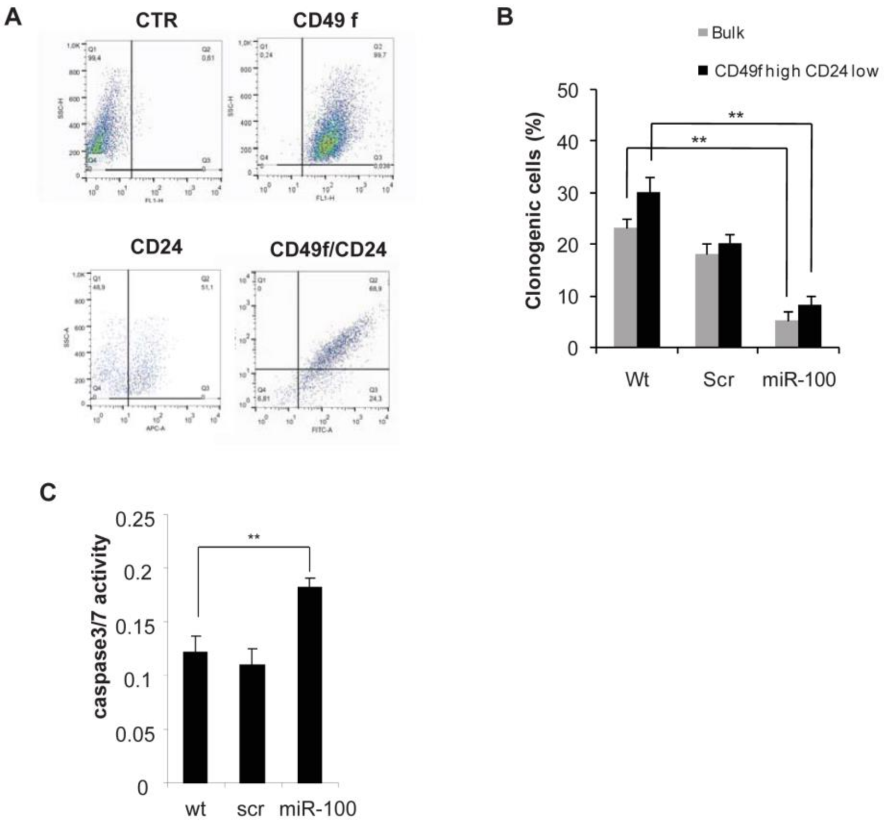

Supplementary Figure 3: MiR-100 affects BrCSC maintenance. A, representative FACS analysis and cell sorting using the CD49f and CD24 markers performed in BrCSCs. **B,** percentage of clonogenicity in bulk and CD49f^{high}/CD24^{low} sorted BrCSCs wild type (wt) and stably expressing either a control scramble (scr) or miR-100. Data are average $\pm$ SD of 3 independent experiments. **C,** analysis of caspase 3/7 activation performed using the Caspase-Glo 3/7 Assay in BrCSCs transduced as in (B). **P<0.01.

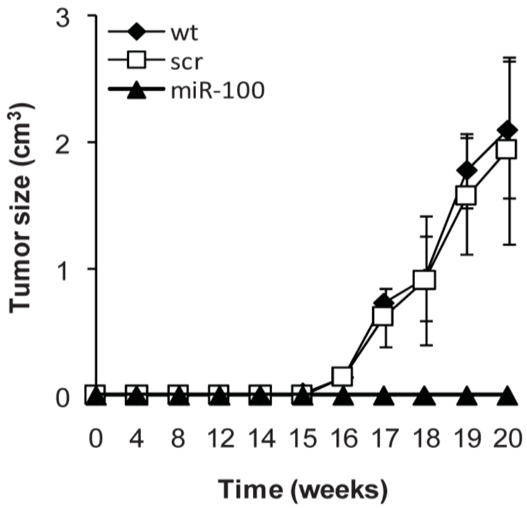

Supplementary Figure 4: MiR-100 affects tumor-initiating ability of BrCSCs. *In vivo* growth of P8 B rCSCs expressed as volume of orthotopic tumors generated by fat-pad injection of either wt, scramble or miR-100 expressing cells. Data are average ± SD of experimental groups containing 6 mice.

Gene name	Fold change	Biological function
ABCB1	-6,1688	ATP-binding cassette, sub-family B
ANGPTL4	-3,0209	growth factrs
CACNA2D3	-3,5105	Calcium Binding and Signaling
CTGF	-2,2217	adhesion
CUBN	-7,119	Calcium Binding and Signaling
DAB2	-3,142	Development & Differentiation:
DLK1	-44,1702	Development & Differentiation:
FOSL1	-3,3211	TF
GDF5	-4,0888	a member of the bone morphogenetic protein (BMP) family
JAG1	-2,5403	notch
LEF1	-19,7667	WNT Signaling
PITX2	-14,74	TF
PLAUR	-2,7734	proteolisis
PPAP2B	-3,0987	calcium signalling
PTGS2	-2,146	calcium signalling
RUNX2	-2,2064	TF
SFRP2	-23,6702	WNT Signaling
T	-6,7975	TF
TCF4	-29,9607	WNT Signaling
WISP1	-42,9623	WNT Signaling
WISP2	-3385,1405	WNT Signaling

CCND2	285,6953	Development & Differentiation:
NRCAM	24,2235	Development & Differentiation:
BMP4	5	Development & Differentiation:

Supplementary Figure 5: MiR-100 inhibits the Wnt/β-catenin signaling pathway. Wnt target gene expression profiling of wild type and miR-100 expressing BrCSCs. In the table, gene expression levels are reported as fold changes between miR-100 expressing and wild type cells. Down-regulated genes are in green, up-regulated genes in red.

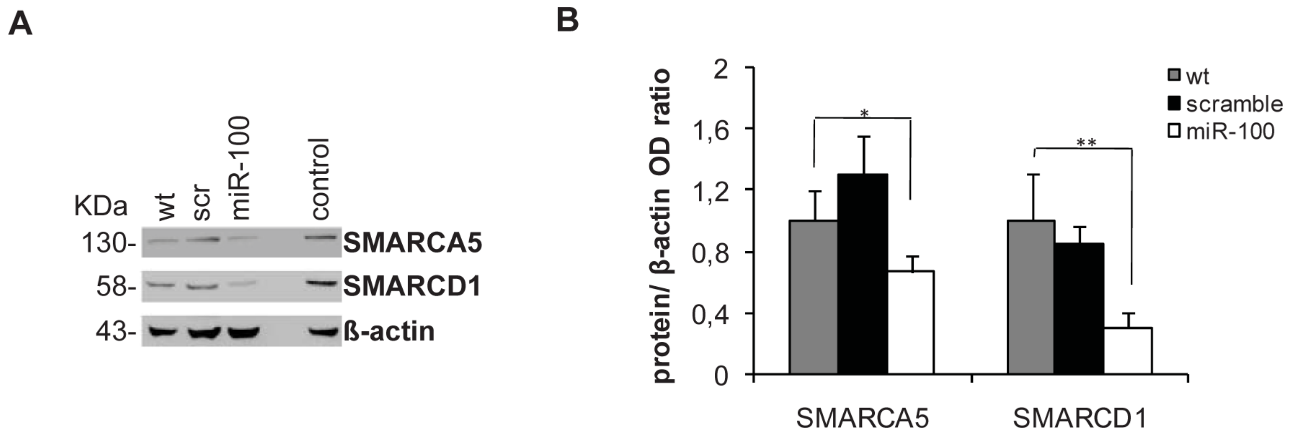

Supplementary Figure 6: MiR-100 downregulates SMARC proteins in BrCSCs. A, Immunoblotting analysis of SMARCA5 and SMARCD1 in wt, scramble or miR-100 expressing BrCSCs; control: total lysate of HeLa cells. **B**, Densitometric quantification of protein expression in BrCSCs transduced as in A. Data represent the mean ± SD of 2 independent experiments. *P<0.05, **P<0.01.

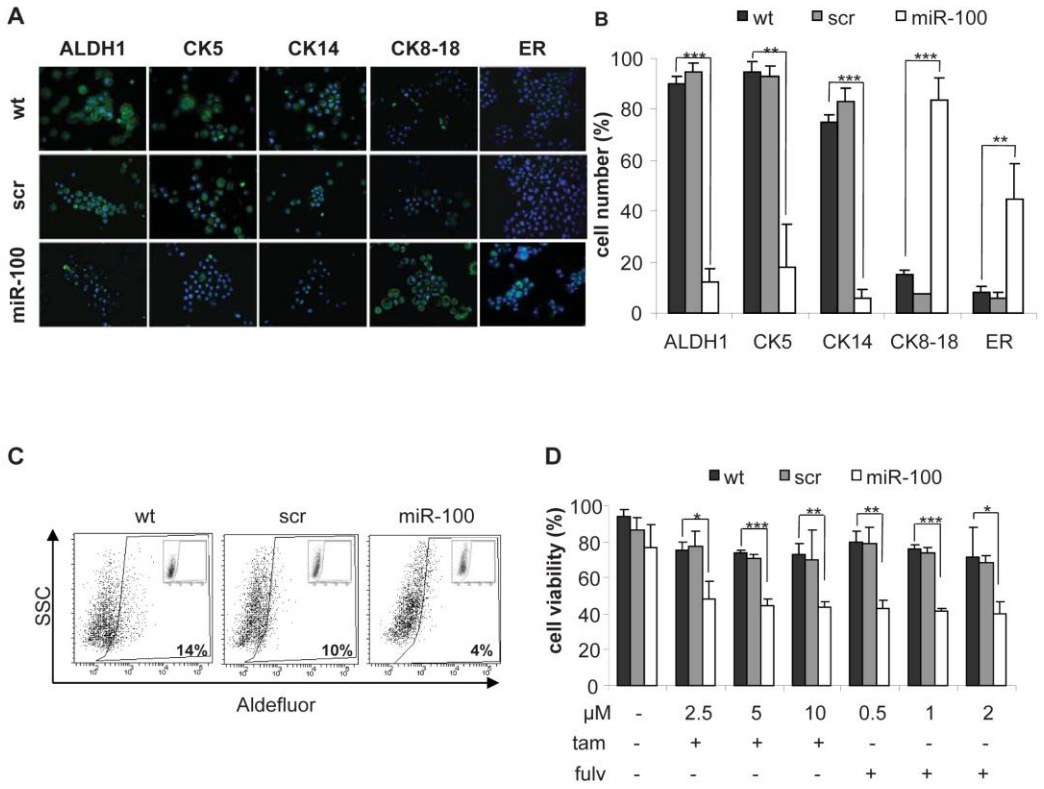

Supplementary Figure 7: Ectopic expression of miR-100 reduces stem cell markers and promotes luminal differentiation in basal-like BrCSCs. A, confocal microscopy analysis of ALDH1, Cytokeratins (CK5, CK14, CK8-18) and ER in patient-derived basal-like BrCSCs (P8), wild type (wt) or stably expressing either a control scramble or miR-100. Nuclei were counterstained by Toto-3 (blue). Magnification 40x. **B,** quantification of the IF staining shown in (A). **C,** flow citometry analysis of Aldefluor assay performed in P8 BrCSCs transduced as in (A). Cells were exposed to Aldefluor substrate (BAAA); cells treated with the specific inhibitor of ALDH1 (DEAB) are shown in the insert panel and were used to define the population with low and high (gated region) ALDH1 activity. **D,** analysis of P8 BrCSC viability upon treatment with tamoxifen (tam) or fulvestrant (fulv) at the indicated doses. *P<0.05, **P<0.01, ***P<0.001.

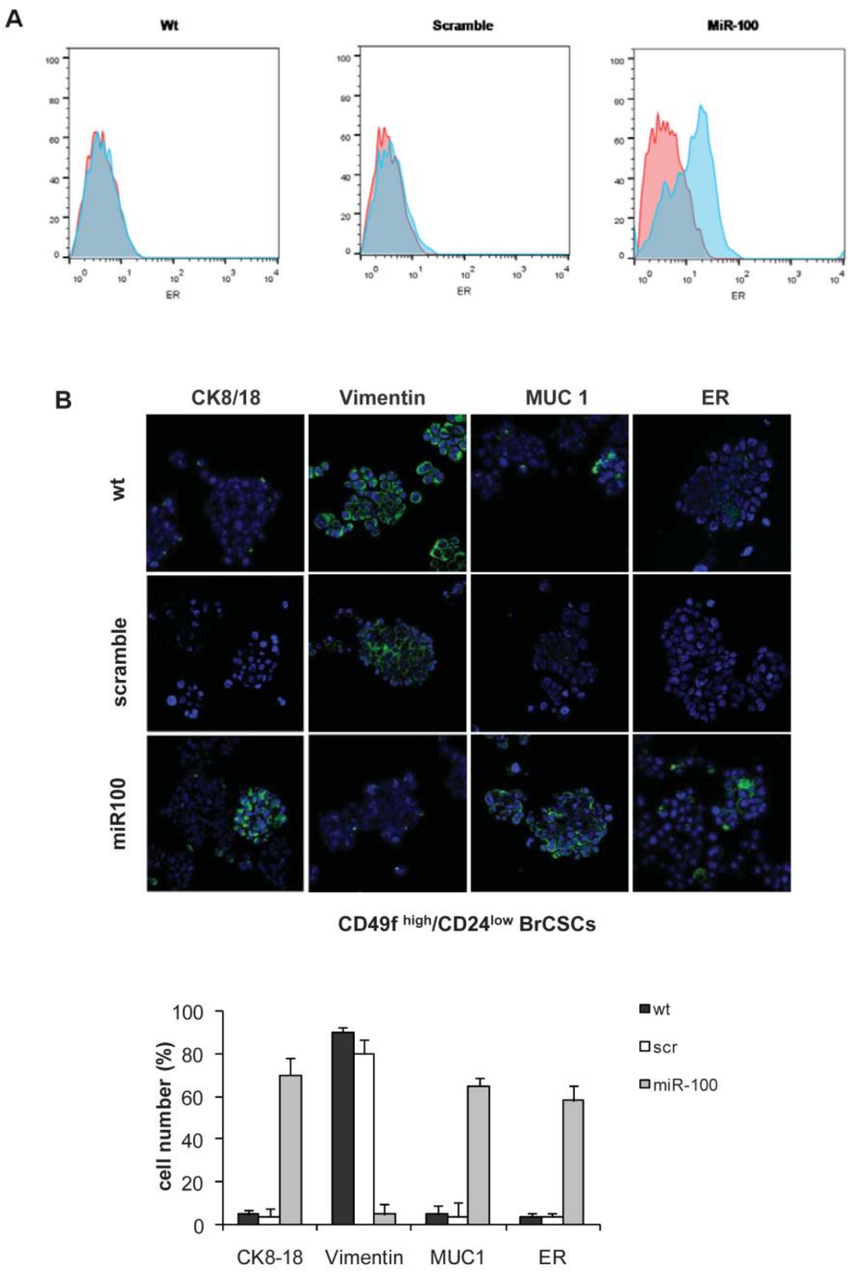

Supplementary Figure 8: Ectopic expression of miR-100 promotes luminal differentiation in CD49f^{high}/CD24^{low} BrCSCs. A, FACS analysis of ER expression in bulk BrCSCs wild type (wt) and stably expressing either a control scramble (scr) or miR-100. **B,** representative confocal microscopy images of IF analysis of CK8-18, Vimentin, MUC1 and ER performed in CD49f^{high}/CD24^{low} BrCSCs wt and stably expressing miR-100. Nuclei were counterstained by Toto-3 (blue). **C**, quantification of the IF staining shown in (B).

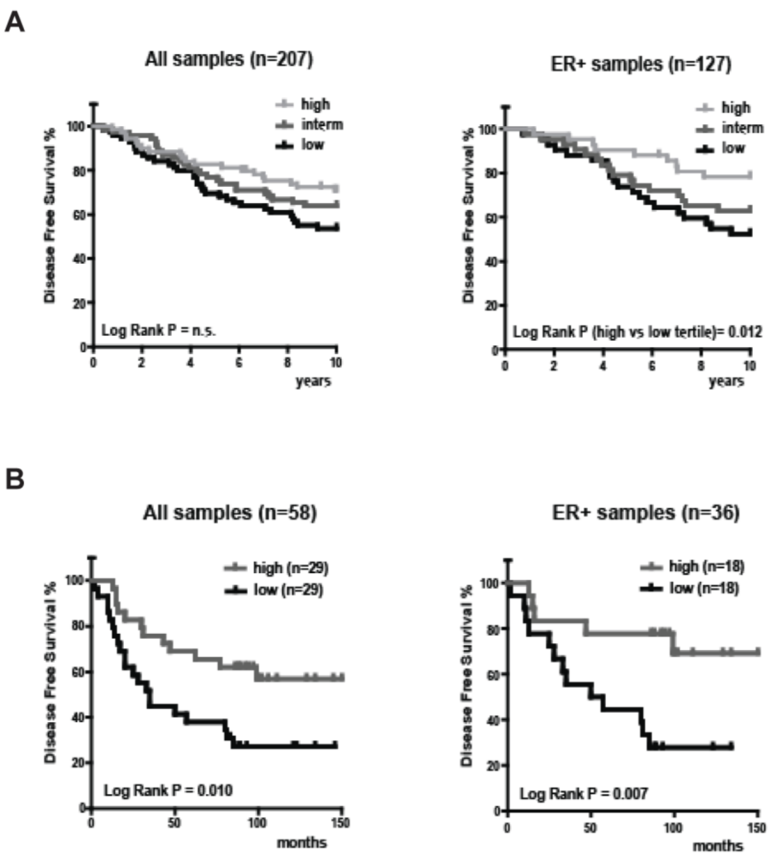

Supplementary Figure 9: Low expression of miR-100 correlates with poor prognosis in two validation cohorts of breast cancer patients. A, Kaplan-Meier curves associated with miR-100 expression in the GEO dataset superSeries GSE22220 (1). Left panel: all patients; right panel: estrogen receptor-positive patients. **B,** Kaplan-Meier curves associated with miR-100 expression in a cohort of 58 patients (for patients' characteristics see Supplementary Table 3). Left panel: all patients; right panel: estrogen receptor-positive patients.

Supplementary Table 1 Case description, tumorigenic capability and $CD44^{+}/CD24^{-}$ analysis

patient	age	Tumor type	grade	ER	PR	HER2	Ki67	Sphere formation	Xenograft	$CD44^{+}/CD24^{-}$
P1	55	IDC	G2	90%	60%	+++	>10%	yes	yes	45%
P2	69	IDC	G2	90%	60%	+	25%	yes	yes	35%
P3	74	IDC	G2	80%	80%	+++	>10%	yes	yes	15%
P4	86	ILC	G2	80%	80%	+++	<10%	yes	yes	65%
P5	85	ILC	G2	-	-	+	>10%	yes	yes	89%
P6	64	IDC	G3	-	-	+++	80%	yes	yes	75%
P7	41	IDC	G3	-	-	-	>30%	yes	no	51%
P8	51	ILC	G2	-	-	+	10-30%	yes	yes	76%

IDC: Infiltrating Ductal Carcinoma, ILC: Infiltrating Lobular Carcinoma

Supplementary Table 2 Characteristics of patients (cohort I)

	**Developing distant metastases**	**Not developing distant metastases**
**Age (years)**		
Range	30-85	37-82
Median	58	52.5
≤ 50	18	27
> 50	41	37
**Size (cm)**		
Range	0.7-9	0.6-4.5
Median	2.2	2
≤ 2	26	38
> 2	32	26
NA	1	0
**Histotype**		
CDI	48	49
CDI+CLI	2	5
CLI	9	3
Other	0	7
**Disease Free Survival (months)**		
Range	8-58	60-185
Median	28	116.5
**ER**		
positive	47	53
negative	12	11
**HER2**		
HER2 positive	9	9
HER2 negative	50	55

Supplementary Table 3 Characteristics of patients (cohort II)

	**Developing distant metastases**	**Not developing distant metastases**
**Age (years)**		
Range	25-79	32-76
Median	51.5	57
≤ 50	14	5
> 50	16	19
NA	3	1
**Size (cm)**		
Range	1.4-8	1-6
Median	3	2.75
≤ 2	6	5
> 2	25	15
NA	2	5
**Histotype**		
CDI	33	25
**Disease Free Survival (months)**		
Range	2-99	83-174
Median	25	102
**Lymphnode status**		
Positive	25	12
negative	8	13
**Grade**		
G1	1	0
G2	18	10
G3	13	12
NA	1	3
**ER**		
positive	18	18
negative	15	7
**PR**		
positive	16	12
negative	14	13
**HER2***		
HER2 positive	6	5
HER2 negative	27	20

* assessed by Real-time analysis
